# Local Hotspots of Endemism or Artifacts of Incorrect Taxonomy? The Status of Microendemic Pill Millipede Species of the Genus *Glomeris* in Northern Italy (Diplopoda, Glomerida)

**DOI:** 10.1371/journal.pone.0162284

**Published:** 2016-09-15

**Authors:** Thomas Wesener, Cathrin Conrad

**Affiliations:** Section Myriapoda, Centre for Taxonomy and Evolutionary Research, Zoological Research Museum A. Koenig, Leibniz Institute for Animal Biodiversity, Bonn, Germany; Sichuan University, CHINA

## Abstract

Local endemic species with their unique evolutionary history always stirred the interest of scientists. One such area especially rich in endemics is northern Italy. In case of pill millipedes of the genus *Glomeris* Latreille, 1803, only a single species is found in northern Europe, while 22 country-endemics alone are known from Italy. Many of these endemics, however, have not been studied in several decades; therefore we aimed to determine whether this diversity is the result of overlooked synonymies or natural processes. A focus was placed on the local endemics that are in some aspects morphologically similar to the widespread and variable *G*. *klugii* Brandt, 1833. The local endemics *Glomeris larii* Verhoeff, 1921, *G*. *primordialis* Verhoeff, 1930, *G*. *oblongoguttata* Verhoeff, 1894, *G*. *oropensis* Verhoeff, 1936, *G*. *transalpina* Koch, 1836, *G*. *romana* Verhoeff, 1900, *G*. *ligurica* Latzel, 1884 and *G*. *apuana* Verhoeff, 1911 were included in a molecular analysis incorporating ribosomal nuclear (28S) and mitochondrial (COI) genes. Individuals were sequenced and compared to 31 specimens from 18 localities of *G*. *klugii*. The final dataset included 657 base pairs for 56 terminals in the COI, and 14 terminals with 1068 base pairs in the combined 28S and COI analysis. Our analysis shows intraspecific distances of up to 5% in the COI gene in *G*. *klugii* that are not strictly correlated to geography or color pattern. *G*. *larii* is discovered to be genetically and morphologically identical to *G*. *klugii* and is synonymised with the latter. Interspecific distances in our dataset vary between 6.7 to 15.9%, with the lowest (6.7–9.0%) between *G*. *primordialis* and *G*. *klugii*. Our analysis confirms the species status of the local endemics *G*. *primordialis*, *G*. *oblongoguttata*, *G*. *oropensis*, *G*. *transalpina*, *G*. *ligurica* and *G*. *apuana*. We also confirm the synonymy of *G*. *undulata* Koch, 1844 under *G*. *klugii*. *G*. *genuensis* Latzel, 1886 is indistinguishable from *G*. *ligurica*.

## Introduction

This study analyses the mitochondrial and nuclear genes of six Italian pill millipede species to determine whether the species are indeed local endemics or simply the result of incorrect taxonomy. Pill millipedes belong to the order Glomerida, a relatively species-poor (>280 species [1]), basal taxon [2] of the Diplopoda with a holarctic distribution [3]. The highest genus-level diversity of the Glomerida is in Europe [1,4]. The most species-rich European genus, *Glomeris* Latreille, 1803 [4,5], includes more than 70 species described from Europe and North Africa [6,7]. Thirty-five of these species are found in Italy and indeed the highest species-level diversity in Europe is reached in Italy just south of the Alps [6,8].

Endemic species are often categorized as microendemics, local endemics, regional endemics, or country endemics. Italy is home to more endemic pill millipede species than in all other European countries combined. Of *Glomeris* species endemic to Italy, 18 occur exclusively in northern Italy [6] and are often only known from a localized area, sometimes just the type locality. Very little is known about these northern Italian endemics, despite their importance for biogeographical studies and nature conservation; fourteen of them have no records since more than 50 years. The species included in our study are six of the 22 endemic *Glomeris* species occurring in Italy [9–11].

The Italian *Glomeris* species studied here all belong historically to the *G*. *klugii* Brandt, 1833 species-group [12,13]. The *G*. *klugii* species-group is a good representative of the potential of molecular studies in helping to clarify taxonomic problems in the genus. Taxonomic progress in *Glomeris* is currently hindered by the unclear status of many of its more than 70 named species, with more than 300 subspecies and varieties being described in the past (see [14]). First molecular studies based on allozyme data settled long suspected synonymies and wrongly applied names in several of the widespread species of the genus [15–18], including *G*. *klugii* [16].

*G*. *klugii* is widespread and is known to show great phenotypic plasticity including many color variations; more than 40 varieties and subspecies have been described [16]. The basic color of *G*. *klugii* is light brown to red, with different amounts of black freckles fusing towards larger spots, and further on towards fully black individuals, creating distinct color patterns [16,19]. Some of the specimens look rather striking, with certain color patterns restricted to special geographic areas, one of the reasons for the rich application of taxonomic names and long list of synonyms in *G*. *klugii*. Two color morphs of *G*. *klugii* were viewed as separate species (*G*. *undulata* Koch, 1844 and *G*. *conspersa* Koch, 1847) for more than 150 years until genetic studies showed their conspecificity [16]. The name *G*. *klugii* Brandt, 1833 was long forgotten and was only recently revised [20]. Its type specimen clearly represents a specimen with the characteristic coloration (red with black spots) of the subspecies *G*. *klugii porphyrea* Koch, 1847 from the Balkan Peninsula. If the subspecies *G*. *k*. *porphyrea* would be a distinct species, the name *G*. *undulata* could be re-validated for the central European populations of *G*. *klugii*.

Modern genetic studies involving single genes, especially the barcoding gene [21,22] cytochrome c oxidase subunit I (COI), can help to further clarify taxonomic problems in pill millipedes. Previous studies show that large genetic p-distances exist between pill millipede genera [23,24]. Large interspecific genetic distances also occur inside the Glomeridae genera, such as in *Trachysphaera* [25] and in *Glomeris* [26], at least between the few species where sequence data is available. First studies based on COI in *Glomeris* revealed two things, (1) local endemic species that were synonyms of more widespread taxa [27]; and (2) 'forgotten' local endemics that could be revised with the help of barcode data [11].

With the help of mitochondrial and nuclear genes, this study evaluates the taxonomic status of six more or less localized endemic Italian *Glomeris* species that historically belong to the *G*. *klugii* species-group: *G*. *larii* Verhoeff, 1921, *G*. *primordialis* Verhoeff, 1930, *G*. *oblongoguttata* Verhoeff, 1894, *G*. *oropensis* Verhoeff, 1936, *G*. *transalpina* Koch, 1836 and *G*. *romana* Verhoeff, 1900. The abovementioned species morphologically resemble the variable and widespread species *G*. *klugii* in the most important morphological characters (coloration and absence of a main stria at the thoracic shield). To clarify the relationships of the local endemics with *G*. *klugii*, specimens of the latter covering the geographical range of the species, as well as representing different color morphs, were included. Our molecular genetic analysis characterizes the different genetic lineages of *G*. *klugii*, as well as shows whether the different local endemic Italian taxa represent valid species or additional synonyms of *G*. *klugii*.

## Materials and Methods

### Specimen selection

**Fig 1:**
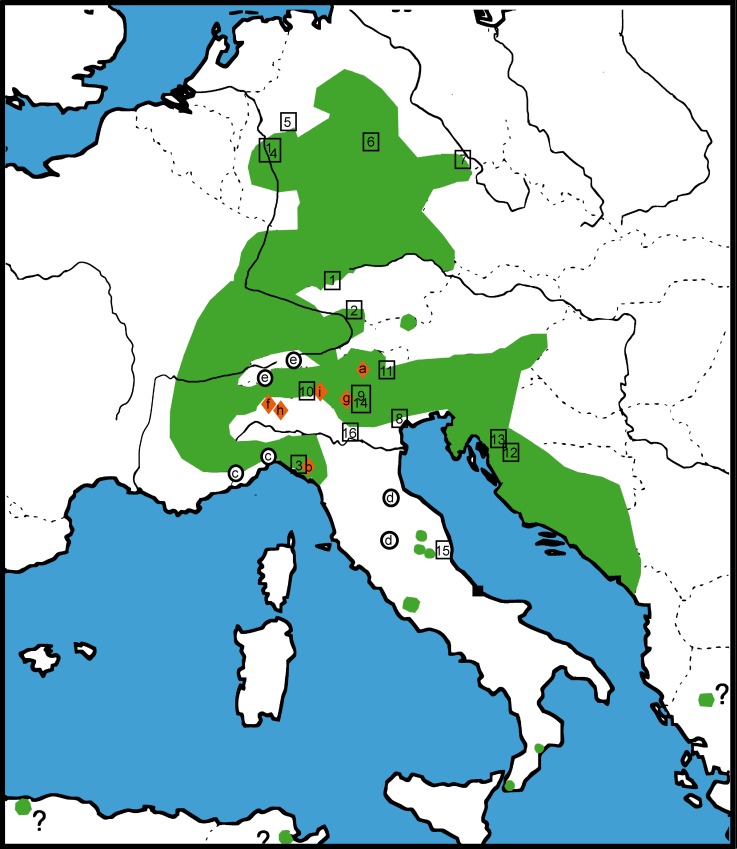
Distribution map of *Glomeris klugii* and sample localities. Known area of distribution of *G*. *klugii* highlighted in green (after Hoess 2000). Letters a-i refer to specific analyzed species (letters marked by a red diamond refer to microendemics), numbers in rectangles to populations (1–16) of *G*. *klugii* (as listed in Table 1).

To study the intraspecific variability of *G*. *klugii* and the taxonomic status of related species we selected 31 specimens of *G*. *klugii* from 18 localities (Fig 1) and downloaded four additional COI sequences of *G*. *klugii* from GenBank (coming from [26]). *Glomeris* species receive no special protection status anywhere in Europe. For specimens collected from natural protection areas, a detailed list of the authority and locations is provided in the acknowledgments. Given the budgetary constraints, we often decided to study only two specimens per locality so that we could include samples from many different populations that represent the whole range and morphological variation of the species. More than two specimens were studied only in especially morphologically diverse populations (Euganean Hills, Hagen, Malgrate).

**Table 1:** Analysed specimens, voucher and Genbank code for the COI analysis. ZFMK = Zoological Research Museum A. Koenig, Bonn, Germany; ZSM = Zoologische Staatssammlung Munich, Germany. Asterisk marks sequences downloaded from Genbank.

Species	Map #	Voucher #	Genbank #, COI/28S	Locality
Glomeris marginata I*	**-**	ZFMK MYR009	FJ409909/KX714022	Germany, Bonn-Kessenich, Venusberg.
G. marginata II*	**-**	ZSM MYR193	HQ966136	Germany, Neustadt (RP), Koenigsbach.
G. connexa I*	**a**	ZSM MYR00372	JN271879	Italy, Lombardia, Sondrio.
G. connexa II*	**-**	ZSM MYR00027	HM888096	Germany, Bavaria, Andechs.
G. connexa III*	**-**	ZSM MYR00028	HM888097	Germany, Bavaria, Scheidegg.
G. connexa IV*	**-**	ZSM MYR00026	HM888095	Germany, Bavaria, Garmisch.
G. connexa V	**-**	ZFMK MYR4232	- /KX714025	Italy, Riva, Valley of W. Riva, under Castanea litter and stones with grasses, 45.86278 N, 010.82240 E, 319 m, 1–2 m right of road, N. slope, coll. 07.iv.2011.
G. apuana I*	**b**	ZFMK MYR752	KT188943	Italy, Liguria, Cinque Terre, SP51 zwischen Volastra und Corniglia, Schluchtwald mit vielen Steinen und Rubus, 510 m, 44°07'34" N, 009°43'33" E, coll. 25.ix.2009.
G. apuana II*	**b**	ZFMK MYR753	KT188944	Italy, Liguria, Cinque Terre, SP51 zwischen Volastra und Corniglia, Schluchtwald mit vielen Steinen und Rubus, 510 m, 44°07'34" N, 009°43'33" E, coll 25.ix.2009.
G. ligurica I*	**c**	ZFMK MYR4440	KT188945/KX714029	Italy, Liguria, between Toirano and Bardineto, San Giorgio di Toirano, 44°10'6.56"N, 8° 9'34.88"E, 811 m, deep ditch very close to pass, 2 m W. of main road, ditch with oak leafs and rubble, coll. 18.iv.2011.
G. ligurica II*	**c**	ZFMK MYR4441	KT188946	Italy, Liguria, between Toirano and Bardineto, San Giorgio di Toirano, 44°10'6.56"N, 8° 9'34.88"E, 811 m, deep ditch very close to pass, 2 m W. of main road, ditch with oak leafs and rubble, coll. 18.iv.2011.
G. ligurica V*	**c**	ZFMK MYR4257	KT188949/KX714030	Italy, Liguria-Piemonte, Ormea, W. of town and river, 44.15039°N, 007.90895°E, 782 m, olive tree cove surrounded by walls, with *Rubus* and *Hedera*, under overgrown stones on wall, coll. 18.iv.2011.
G. romana marinensis	**d**	ZFMK MYR797	LX714036/KX714026	San Marino, San Marino Stadt, Wiese unter Steinen, nahe Seilbahnstation Tal, leg. 16.ix.2009.
G. romana romana	**d**	ZFMK MYR1471	KX714037	Italy, Umbria, Perugia, NE Gosparini, Dry *Quercus* forest, dired-out stream with water puddles, 43°14'31.73" N, 12°06'19.69" E, 550 m, 29.viii.2012.
G. transalpina I	**e**	ZFMK MYR2609	KX714038	Switzerland, Wallis, Riederalm, E. Riederfurka, 2170 m, 46°22'57.19" N, 8°1'20.45" E, overgrown rock and under dead wood, leg. 22.vi.2014.
G. transalpina II	**e**	ZFMK MYR2636	KX714039	Switzerland, Wallis, Simplonpass, 2130 m, 46°14'50.46"N, 8°2'19.88"E, Bachtal mit überwachsenen Felsen, under *Rhododendron*, leg. 23.vi.2014.
G. oropensis	**f**	ZFMK MYR4534	KX714040/KX714028	Italy, Piemonte, Biella, NW Sanctuary of Oropa, 45.62947°N, 007.98168°E, 1200 m, Fagus forest with stones, coll. 14.iv.2011.
G. oblongoguttata I	**g**	ZFMK MYR4567	KX714041/KX714027	Italy, Lago di Iseo, Pisogne, 45.79851°N, 010.11522°E, 281 m, quarry with lots of soft calcareous stones, sparse *Populus* only vegetation, under stones.
G. oblongoguttata II	**g**	ZFMK MYR4570	KX714042	Italy, Lago di Iseo, Pisogne, 45.79851°N, 010.11522°E, 281 m, quarry with lots of soft calcareous stones, sparse *Populus* only vegetation, under stones.
G. oblongoguttata III	**g**	ZFMK MYR4564	KX714043	Italy, Lago di Iseo, Pisogne, 45.79851°N, 010.11522°E, 281 m, quarry with lots of soft calcareous stones, sparse *Populus* only vegetation, under stones.
G. oblongoguttata IV	**g**	ZFMK MYR4569	KX714044	Italy, Lago di Iseo, Pisogne, 45.79851°N, 010.11522°E, 281 m, quarry with lots of soft calcareous stones, sparse *Populus* only vegetation, under stones.
G. oblongoguttata V	**g**	ZFMK MYR4568	KX714045	Italy, Lago di Iseo, Pisogne, 45.79851°N, 010.11522°E, 281 m, quarry with lots of soft calcareous stones, sparse *Populus* only vegetation, under stones.
G. primordialis I	**h**	ZFMK MYR4744	KX714046/KX714023	Italy, Piemonte, Biella, Pollone—Favaro, 'Via per Oropa', 45.58936°N, 008.00297°E, 626 m, Small stream valley with light understory and young *Fraxinus*, *Castanea*, *Acer* vegetation, coll. 13.iv.2011.
G. primordialis II	**h**	ZFMK MYR4741	KX714047	Italy, Piemonte, Biella, Pollone—Favaro, 'Via per Oropa', 45.58936°N, 008.00297°E, 626 m, Small stream valley with light understory and young *Fraxinus*, *Castanea*, *Acer* vegetation, coll. 13.iv.2011.
G. primordialis III	**h**	ZFMK MYR4745	KX714048/KX714024	Italy, Piemonte, Biella, Pollone—Favaro, 'Via per Oropa', 45.58936°N, 008.00297°E, 626 m, Small stream valley with light understory and young *Fraxinus*, *Castanea*, *Acer* vegetation, coll. 13.iv.2011
G. larii	**i**	ZFMK MYR4562	KX714049/KX714031	Italy, Lago di Como, Malgrate, Bocca, 45.84660°N, 009.38021°E, 282 m, NE. slope with numerous stones (calcareous and kiesel), few *Castanea* and *Acer*, under stones, in leaf litter, between both walkways, coll. 10.iv.2011.
G. klugii I*	**1**	ZSM MYR00192	HQ966135	Germany, Bavaria, Solnhofen.
G. klugii II*	**2**	ZSM MYR00040	HM888106	Germany, Bavaria, Scheidegg.
G. klugii CQIIa*	**3**	ZFMK MYR4444a	KT188951	Italy, Liguria, Cinque Terre, SP51 zwischen Volastra und Corniglia, Schluchtwald mit vielen Steinen und Rubus, 510 m, 44°07'34" N, 009°43'33" E, coll. 25.ix.2009
G. klugii CQIIb*	**3**	ZFMK MYR4444b	KT188952	Italy, Liguria, Cinque Terre, SP51 zwischen Volastra und Corniglia, Schluchtwald mit vielen Steinen und Rubus, 510 m, 44°07'34" N, 009°43'33" E, coll. 25.ix.2009
G. klugii CC105	**4**	ZFMK MYR4768	KX714060	Germany, Siebengebirge, Löwenburg, between Olender and Lohrberg, old Roman quarry, left of path, Acer-Fraxinetum, 50°40'02"N, 7°14'17"E, coll. 21.09.2011.
G. klugii CC104	**4**	ZFMK MYR4769	KX714061/KX714032	Germany, Siebengebirge, Löwenburg, between Olender and Lohrberg, old Roman quarry, left of path, Acer-Fraxinetum, 50°40'02"N, 7°14'17"E, coll. 21.09.2011.
G. klugii CC011	**5**	ZFMK MYR4521	KX714056	Germany, NRW, Hagen-Holthausen, Weißenstein, 174 m, 51°21'39.72"N, 7°32'59.79"E, NE slope with numerous calcareus stones and soft Lößboden, coll. 20.v.2011.
G. klugii CC115	**6**	ZFMK MYR4775	KX714055	Germany, Thuringia, Bad Langensalza, Hainich, Thiumsburg, Fagus forest. 51.081°N, 10.128°E, coll. 05.ix.2011.
G. klugii CC004	**2**	ZFMK MYR815	KX714053	Germany, Bavaria, 1 km WSW Scheidegg, 780 m, Fauna Bavarica, J. Spelda
G. klugii CC001	**7**	ZFMK MYR740	KX714054	Germany, Saxony, Dohnatal, Weesenstein, Hangwald, unter Steinen, 50°55.50' N, 13°51.38' E, leg. 24.x.2009
G. klugii CC012	**5**	ZFMK MYR4522	KX714057	Germany, NRW, Hagen-Holthausen, Weißenstein, 174 m, 51°21'39.72"N, 7°32'59.79"E, NE slope with numerous calcareus stones and soft Lößboden, coll. 20.v.2011.
G. klugii CC013	**5**	ZFMK MYR4520	KX714058	Germany, NRW, Hagen-Holthausen, Weißenstein, 174 m, 51°21'39.72"N, 7°32'59.79"E, NE slope with numerous calcareus stones and soft Lößboden, coll. 20.v.2011.
G. klugii CC016	**5**	ZFMK MYR4519	KX714059/KX714033	Germany, NRW, Hagen-Holthausen, Weißenstein, 174 m, 51°21'39.72"N, 7°32'59.79"E, NE slope with numerous calcareus stones and soft Lößboden, coll. 20.v.2011.
G. klugii CC039	**8**	ZFMK MYR4734	KX714072	Italy, Euganean Hills, valley on NW foot of Monte Venda, 45.31448°N, 011.66336°E, 187 m, NNE facing slope under *Castanea* leafs and stones, coll. 05.iv.2011.
G. klugii CC042	**8**	ZFMK MYR4737	KX714074	Italy, Euganean Hills, valley on NW foot of Monte Venda, 45.31448°N, 011.66336°E, 187 m, NNE facing slope under *Castanea* leafs and stones, coll. 05.iv.2011.
G. klugii CC025	**9**	ZFMK MYR4729	KX714065	Italy, Riva, Valley of W. Riva, under *Castanea* leaves and stones with grasses, 45.86278° N, 010.82240° E, 319 m, 1–2 m right of road, N. slope, coll 07.iv.2011.
G. klugii CC024	**9**	ZFMK MYR4728	KX714066	Italy, Riva, Valley of W. Riva, under *Castanea* leaves and stones with grasses, 45.86278° N, 010.82240° E, 319 m, 1–2 m right of road, N. slope, coll 07.iv.2011.
G. klugii CC040	**8**	ZFMK MYR4735	KX714073/KX714034	Italy, Euganean Hills, valley on NW foot of Monte Venda, 45.31448°N, 011.66336°E, 187 m, NNE facing slope under *Castanea* leafs and stones, coll. 05.iv.2011.
G. klugii CC092	**10**	ZFMK MYR4560	KX714069	Italy, Lago di Como, Malgrate-Pare (Malgrate), 45.84782°N, 009.38142°E, 242 m, steep NW slope with *Hedera* and grass, under stones, 1–3 m S of road, coll. 10.iv.2011.
G. klugii CC096	**10**	ZFMK MYR4766	KX714070	Italy, Lago di Como, Malgrate-Pare (Malgrate), 45.84782°N, 009.38142°E, 242 m, steep NW. slope with *Hedera* and grass, under stones, 1–3 m S of road, coll. 10.iv.2011.
G. klugii CC093	**10**	ZFMK MYR4559	KX714068	Italy, Lago di Como, Malgrate-Pare (Malgrate), 45.84782°N, 009.38142°E, 242 m, steep NW. slope with *Hedera* and grass, under stones, 1–3 m S of road, coll. 10.iv.2011.
G. klugii CC101	**10**	ZFMK MYR4767	KX714067	Italy, Lago di Como, Malgrate, Bocca, 45.84660°N, 009.38021°E, 282 m, NE. slope with numerous stones (calcareous and kiesel), few *Castanea* and *Acer*, under stones, in leaf litter, between both walkways, coll. 10.iv.2011.
G. klugii CC111	**11**	ZFMK MYR4771	KX714062	Italien, Südtirol, Road between Aguai (SS48) and Altrei, Abies-rich forest valley, SW slope, 46°17'35.06"N, 11°23'35.72"E, 1218 m, coll. 24.ix.2011.
G. klugii CC110	**11**	ZFMK MYR4770	KX714063	Italien, Südtirol, Road between Aguai (SS48) and Altrei, Abies-rich forest valley, SW slope, 46°17'35.06"N, 11°23'35.72"E, 1218 m, coll. 24.ix.2011.
G. klugii porphyrea CC007	**12**	ZFMK MYR734	KX714052	Kroatien, Pilwitzer Seen, Plitvicka Jezera, oberhalb der Seen, 44.8762° N, 15.6309° E, 597 m, Karst mit tiefen Dolinen, Buchenwald, im Laub, leg. 13.x.2009
G. klugii porphyrea CC005	**13**	ZFMK MYR4523	KX714050	Kroatien, Velika Kapela, Weg zwischen Alan und Dreznica, 45.1026° N, 14.9658° E, 890 m, Gebirgswald Tanne-Bergahorn, unter Nadeln und Steinen am Wegesrand, leg 16.x.2009
G. klugii porphyrea CC006	**13**	ZFMK MYR4524	KX714051	Kroatien, Velika Kapela, Weg zwischen Alan und Dreznica, 45.1026° N, 14.9658° E, 890 m, Gebirgswald Tanne-Bergahorn, unter Nadeln und Steinen am Wegesrand, leg. 16.x.2009
G. klugii CC023	**14**	ZFMK MYR4590	KX714064	Italy, Lago di Garda, Salo, Renzano, 45.628545°N, 010.52181°E, 399 m, N. slope, under leaf litter and vegetation, coll. 03.iv.2011.
G. klugii CC103	**15**	ZFMK MYR111	KX714075	Italy, Abruzzen, Chieti, Fara Filiorum Petri, Flußaue mit nährstoffreichem Lehmboden, unter Holz und Steinen, 211 m, 42°14'46.5'' N, 14°11'5.6'' E, leg. 5.iv.2006.
G. klugii TW99	**16**	ZFMK MYR4260	KX714071	Italy, Lombarida, Cremona, 45.313711° N, 9.878526° E, leg. 02.2014.
G. klugii CC107	**3**	ZFMK MYR637	KX714076/KX714035	Italy, Liguria, Cinque Terre, SP51 between Volastra and Corniglia, Schluchtwald mit vielen Steinen und Rubus, 510 m, 44°07'34" N, 009°43'33" E, leg. 25.ix.2009

For the study of the northern Italian local endemic species, we selected specimens of *G*. *larii*, *G*. *primordialis*, *G*. *oblongoguttata*, *G*. *oropensis*, *G*. *transalpina*, *G*. *romana*, *G*. *ligurica* Latzel, 1884 and *G*. *apuana* Verhoeff, 1911; the sequences of *G*. *ligurica* and *G*. *apuana* came from earlier studies [11]. Most of the local endemic species are rare, meaning that only a few specimens, often from only a single locality, could be included.

We also selected specimens of *G*. *marginata* and *G*. *connexa* to serve as outgroups for the study of the 28S rDNA gene as no pill millipede sequences were available on Genbank (06.2015).

All specimens were directly conserved in 95% ethanol and then, within days or weeks, stored at -20°C until DNA extraction.

### DNA extraction, PCR, and sequencing

For DNA extraction, we removed inter-segmental muscle tissue from the specimens and then, following the manufacturer’s extraction protocol, extracted genomic DNA from muscle tissue using the DNAeasy Blood & Tissue kit from Qiagen.

Voucher specimens of each sequenced animal are stored at the ZFMK (Table 1). PCR and sequencing success for the COI gene was low; several iterations of PCRs and sequencing reactions were necessary.

We conducted the PCR of the COI gene with the LCO1490 and Nancy primer pair [28], as well as the Folmer primers [29]. Each reaction involved 2.5 μl DNA, 2.3 μl water, 2 μl Q-solution, 10 μl Qiagen multiplex-mix, and 1.6 μl of each primer (10 pmol/μl) for a total reaction volume of 20 μl. Each PCR run included a negative control without any DNA. The touchdown PCR temperature profile was 35 seconds of denaturation at 94°C, 90 seconds annealing at 55°C (40°C), 90 seconds of elongation at 72°C at 15 (25) cycles. Of 112 samples, only 41 were successfully amplified. Several samples of *G*. *klugii* did not yield any PCR bands. This was similar to the results of a previous study (Spelda et al. 2011). Studies using a more degenerated primer pair had a much higher success rate in *Glomeris* species (see [11,27,30].

For the 28S analysis, we studied the D3-D5 fragment, using primers successfully utilized in previous studies of millipedes [31]. Each reaction involved 2 μl DNA, 2.8 μl water, 2 μl Q-solution, 10 μl Qiagen multiplex-mix, and 1.6 μl of each primer (10 pmol/μl) for a total reaction volume of 20 μl. Each PCR run included a negative control without any DNA. The PCR temperature profile was 60 seconds of denaturation at 94°C, 60 seconds annealing at 50.5°C, 60 seconds of elongation at 72°C at 35 cycles. The PCR was successful for all 14 samples.

Subsequent Sanger sequencing of purified PCR-products was outsourced and conducted with the same primers at Macrogen, Netherlands. We confirmed sequence identities with BLAST searches [32], checked our data for multi-peaks, translated sequences into amino-acids, and checked whether nucleotide substitutions were primarily found at the third codon position to rule-out pseudogenes [33]. All 41 new COI sequences (2x *G*. *romana*, 2x *G*. *transalpina*, 1x *G*. *oropensis*, 5x *G*. *oblongoguttata*, 3x *G*. *primordialis*, 1x *G*. *larii*, 27x *G*. *klugii*), as well as all 14 new 28S sequences (1x *G*. *larii*, 4x *G*. *klugii*, 1x *G*. *oropensis*, 1x *G*. *oblongoguttata*, 2x *G*. *primordialis*, 1x *G*. *romana*, 1x *G*. *marginata*, 1x *G*. *connexa*, 2x *G*. *ligurica*) were deposited in GenBank (see Table 1 for access numbers).

### Phylogenetic and distance analyses

The COI analysis included all species and specimens that could be sequenced (see above). Double strand sequences were concatenated in SEQMAN and aligned by hand in Bioedit [34]. Evolutionary analyses were conducted in MEGA6 [35]. For the analysis of the COI gene, the evolutionary history was inferred by using the Maximum Likelihood method based on the Hasegawa-Kishino-Yano model [36]. The tree with the highest log likelihood (-3621.8103) is shown. The percentage of trees in which the associated taxa clustered together is shown next to the branches. Initial tree(s) for the heuristic search were obtained automatically by applying Neighbor-Join and BioNJ algorithms to a matrix of pairwise distances estimated using the Maximum Composite Likelihood (MCL) approach, and then selecting the topology with superior log likelihood value. A discrete Gamma distribution was used to model evolutionary rate differences among sites (5 categories (+G, parameter = 0.7657)). The rate variation model allowed for some sites to be evolutionarily invariable ([+I], 60.6677% sites). The tree is drawn to scale, with branch lengths measured in the number of substitutions per site. The analysis involved 56 nucleotide sequences. Codon positions included were 1st+2nd+3rd+Noncoding. There were a total of 657 positions in the final dataset.

Aside from *G*. *klugii*, the 28S analysis included specimens of the local endemic species *G*. *larii*, *G*. *primordialis*, *G*. *oblongoguttata*, *G*. *oropensis*, *G*. *ligurica* and *G*. *romana*, as well as the most widespread *Glomeris* taxa (after [6]), *G*. *marginata* and *G*. *connexa* as outgroups. Budgetary constraints did not allow for a broader analysis. Successfully amplified and sequenced were *G*. *klugii* specimens from the Cinque Terre (W Italy), the Euganean Hills (E Italy), Hagen (NW Germany) and the Siebengebirge (W Germany), which cover the geographical range of the species. Double strand sequences were concatenated with Seqman (DNASTAR Inc.). Noisy ends were filled-in with 'N's. The dataset was aligned with MUSCLE [37]. The uncut 28S data set had 425 positions. Because of an unclear homology, positions 170–183 were cut before further analyses were conducted, resulting in a dataset with 411 positions.

Because the 28S dataset showed extremely little resolution on the here analyzed level (maximum distances 2.3%), COI sequences from the same specimens were added to concatenate the dataset.

For the combined dataset, the evolutionary history was inferred by using the Maximum Likelihood method the same way as in the COI analysis (see above). A discrete Gamma distribution was used to model evolutionary rate differences among sites (5 categories (+G, parameter = 0.1627)). The rate variation model allowed for some sites to be evolutionarily invariable ([+I], 38.1750% sites). The tree is drawn to scale, with branch lengths measured in the number of substitutions per site. The analysis involved 14 nucleotide sequences. There were a total of 1068 positions in the final dataset (S1 Alignment).

For both the analysis of the COI gene and the analysis of the combined dataset, a bootstrap consensus tree inferred from 1000 replicates [38] is taken to represent the evolutionary history of the analyzed taxa.

For the distance analysis, only the COI dataset was used. Uncorrected p-distances were calculated using MEGA6 [35]. The number of base differences per site from between sequences are shown (S1 Table). The analysis involved 56 nucleotide sequences. Codon positions included were 1st+2nd+3rd+Noncoding. All ambiguous positions were removed for each sequenced pair. There were a total of 657 positions in the final dataset.

## Results

### The genetic distance analysis

**Fig 2:**
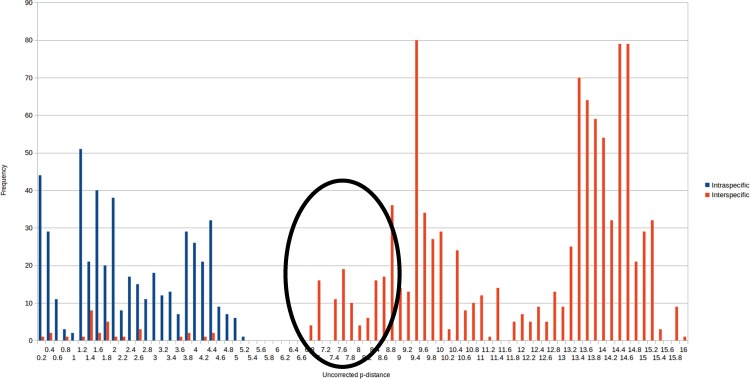
Frequency distribution of pairwise intraspecific (blue) and interspecific (red) distances. All red points in the blue area refer to *Glomeris larii*. Basic table see S1 Table.

Aside from *G*. *larii*, interspecific distances in our COI dataset vary between 6.7–16.1%, while intraspecific distances are between 0–5% (Fig 2). A clear barcoding gap is present between 5% and 6.7% (Fig 2), which rises well above 8.5% if one species (*G*. *primordialis*) is removed.

*G*. *larii*, described as an endemic of the Lake Como area, shows little to no genetic distance in the COI barcoding fragment to *G*. *klugii* specimens from the same locality (0.2%, or a single basepair) and a small distance of 0.3–4.4% to the other populations of *G*. *klugii*, falling clearly inside the observed intraspecific variation of *Glomeris* species (<5%, see Fig 2).

*G*. *primordialis* differs 6.7–9% from *G*. *klugii*. The three sequenced specimens of *G*. *primordialis* show an intraspecific variation of 0.8–2.6%.

*G*. *oblongoguttata* differs from its closest relatives, *G*. *klugii* and *G*. *transalpina*, by 9.1–10.4% and 10.8–11.4% respectively. Intraspecific variation of the five analyzed *G*. *oblongoguttata* specimens, all from the same population, is low, 0–0.3%.

*G*. *oropensis* clearly differs from the two nearby populations of *G*. *transalpina* by 9.9%, from *G*. *primordialis*, which occurs almost in sympatry, by 9.4–10.1%, and by 9.3–11.7% from the widespread *G*. *klugii*.

*G*. *transalpina* shows its lowest genetic distances to *G*. *klugii*, with genetic distances of 8.4–9.4%. Both sequenced *G*. *transalpina* populations show identical haplotypes (0% distance).

*G*. *romana* shows large genetic distances to all other analyzed species, varying between 13.1–15.4%. The two analyzed subspecies, *G*. *romana romana* Verhoeff, 1900 and *G*. *romana marinensis* Verhoeff, 1928 differ at a single basepair.

Distances between the species *G*. *marginata*, *G*. *connexa*, *G*. *apuana* and *G*. *ligurica* are high, always >10%, while their intraspecific distances are between 0–2.0%

### Phylogeny of the *Glomeris* species

**Fig 3:**
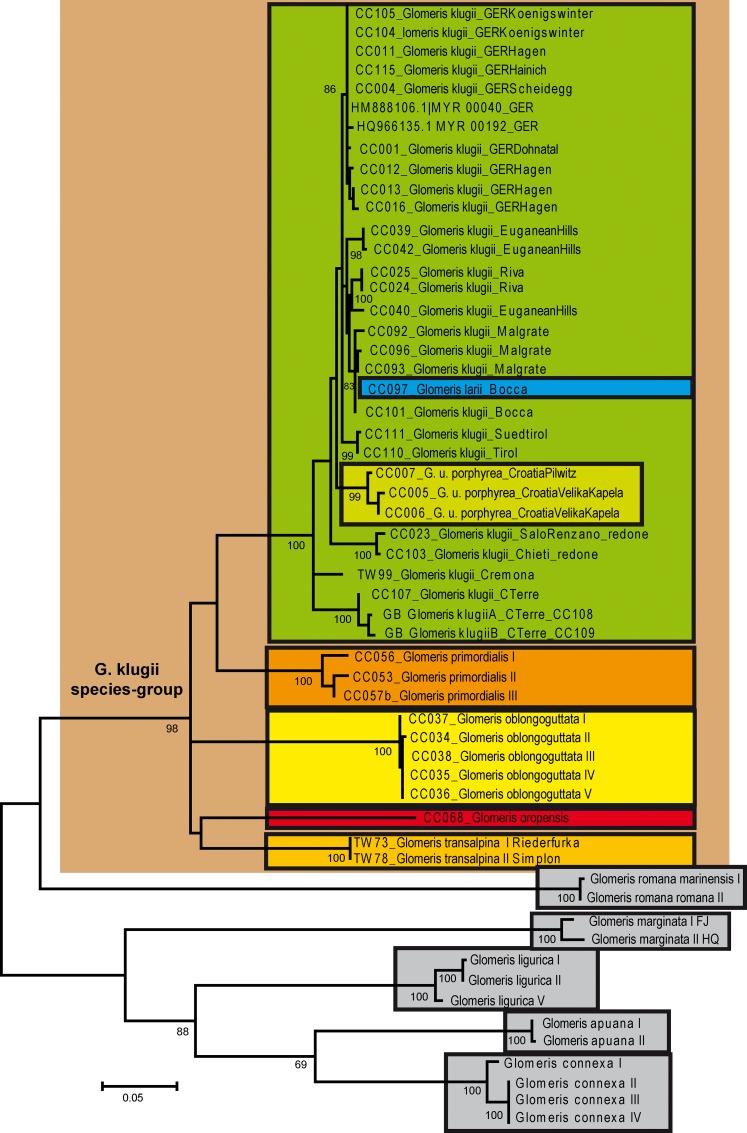
Phylogenetic tree recovered in the maximum likelihood analysis based on the COI gene. Numbers represent nodal support values from the maximum likelihood (1000 bootstrap replicates) analysis. Values >65% not shown. Sequences from GenBank marked with single asterisk after name. Species and subspecies surrounded by boxes. *G*. *klugii* species-group highlighted by brown background.

**Fig 4:**
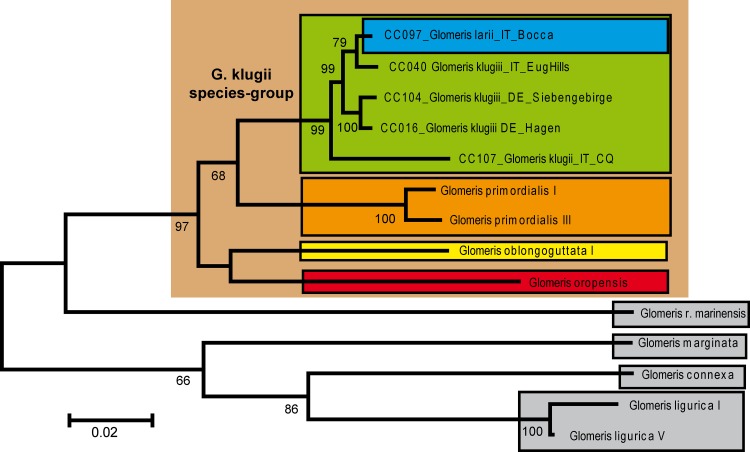
Phylogenetic tree recovered in the combined maximum likelihood analysis based on the 28S and COI gene. Numbers represent nodal support values from the maximum likelihood (1000 bootstrap replicates) analysis. Values <65% not shown. Sequences from GenBank marked with single asterisk after name. Species and subspecies surrounded by boxes. *G*. *klugii* species-group highlighted by brown background.

The phylogeny of the analyzed *Glomeris* species was resolved congruently in both the COI (Fig 3) and the combined dataset (Fig 4). Because the COI dataset included more taxa, it is here used for further discussions. The *G*. *klugii* species-group excluding *G*. *romana* is recovered as monophyletic with strong statistical support (98%). Inside the *G*. *klugii* species-group, no species grouping receives statistical support. A basal trichotomy includes a grouping uniting the high-alpine endemics *G*. *transalpina* and *G*. *oropensis*, the Bergamasque endemic (Fig 1) *G*. *oblongoguttata*, and a grouping uniting *G*. *primordialis* and *G*. *klugii*, with *G*. *larii* nested deeply inside *G*. *klugii*. The monophyly of each species (except *G*. *larii*) is statistically strongly supported (100%). The grouping of *G*. *larii* inside *G*. *klugii* is statistically strongly supported (100%).

The monophyly of the other analyzed *Glomeris* species, *G*. *romana*, *G*. *marginata*, *G*. *ligurica*, *G*. *apuana* and *G*. *connexa*, is strongly supported (all 100%). The only supported grouping (Fig 3) is the *G*. *connexa* species-group (88%), including *G*. *ligurica*, a western Italian endemic (Fig 1: c), in a basal position opposed to the weakly-supported (69%) sister-taxa *G*. *connexa* and *G*. *apuana*.

### Color morphs and geographical variation in *G*. *klugii*

The characteristically colored subspecies *G*. *klugii porphyrea* from the Balkan Peninsula is nested within *G*. *klugii* (Fig 3) and shows genetic distances to the other populations of the species in the COI barcoding fragment of 1.9–5.0%. These Balkan specimens do not form a monophylum with other similarly colored specimens from Italy (Fig 2).

Inside *G*. *klugii*, the resolution of the COI (Fig 3) and combined (Fig 4) analyses is limited. In both analyses the specimens from Liguria, collected around the Cinque Terre (Fig 1: 3), are the most-basal specimens, opposed, but statistically not supported, to the remaining specimens. The unusually colored population from Cremona in the Po Valley (Fig 1: 16) is in a basal trichotomy with the specimens from the Cinque Terre and all remaining *G*. *klugii*. The next split /includes two specimens (100% bootstrap support) from disjunct populations, one a red-black specimen from the west coast of the Lago di Garda (Fig 1: 14), the second a normal colored specimen from Chieti, located in the southern margin of the species range (Fig 1: 15). The following split (99% statistical support) includes the specimens of the subspecies *G*. *klugii porphyrea* from two sites in Croatia at the eastern edge of the range of the species. The next split opposes two specimens from Southern Tyrol, Italy (Fig 1: 11) and the German and remaining Italian *G*. *klugii* (Fig 4). A further split then separates all German specimens from the remaining specimens from Italy (Fig 3). All German specimens group together with moderate statistical support (86%). All seven sampled German populations, spanning the northern and NE margin of the species range, show genetic distances of 0–0.6% from one another, with up to 0.3% observed in the same population (Hagen, four specimens sampled), with the same haplotype present in geographically close populations.

For the remaining Italian populations, the resolution is limited (Fig 4). The genetic distances in the group are moderate, 0–2.5%. Two specimens from the Euganean Hills (Fig 1: 8) are basally located, while a third specimen from the same population groups with two specimens from Riva, which is on the northern coast of the Lago di Garda (Fig 1: 9). The specimens from Malgrate and Bocca, two sites near Lecco on the Lago di Como (Fig 1: 10), group together with the specimen of *G*. *larii* from the same area (Fig 4).

## Discussion

### Intraspecific differences in *G*. *klugii*

The observed 5% intraspecific distances within *G*. *klugii* are high in comparison to other *Glomeris* species, which usually fall below 2.5% [26] and 3.5% [27]. However, all previous studies included less than 10 specimens per species and never covered a large geographic area. For example, barcoding studies of German ground beetles (Carabidae) showed intraspecific distances of up to 3.8% in the COI gene, weakly overlapping with interspecific distances which showed a minimum 3% p-distance [39]. Other Europe-wide scale studies focusing on rose beetles found intraspecific distances of up to 9% [40].

The present study explicitly included *G*. *klugii* specimens from the extremes of the species’ northern, eastern and southern range. If we included only German populations in our analysis, intraspecific variation would be at up to 0.5% only. The highest intraspecific distances were observed in the Italian *G*. *klugii*. In Italy, no geographic pattern of the distribution of related haplotypes could be detected. For example, a specimen (CC023) from Salo is more closely related to a specimen from Chieti (Fig 3) than to a nearby population from Riva (Fig 1). Also, different, unrelated haplotypes exist in the same Italian populations of *G*. *klugii*, as is evident by the three analyzed specimens from the Euganean Hills (Fig 1). Two of them are close to one another, while a third specimen groups more closely to specimens from Riva (Fig 3).

**Fig 5:**
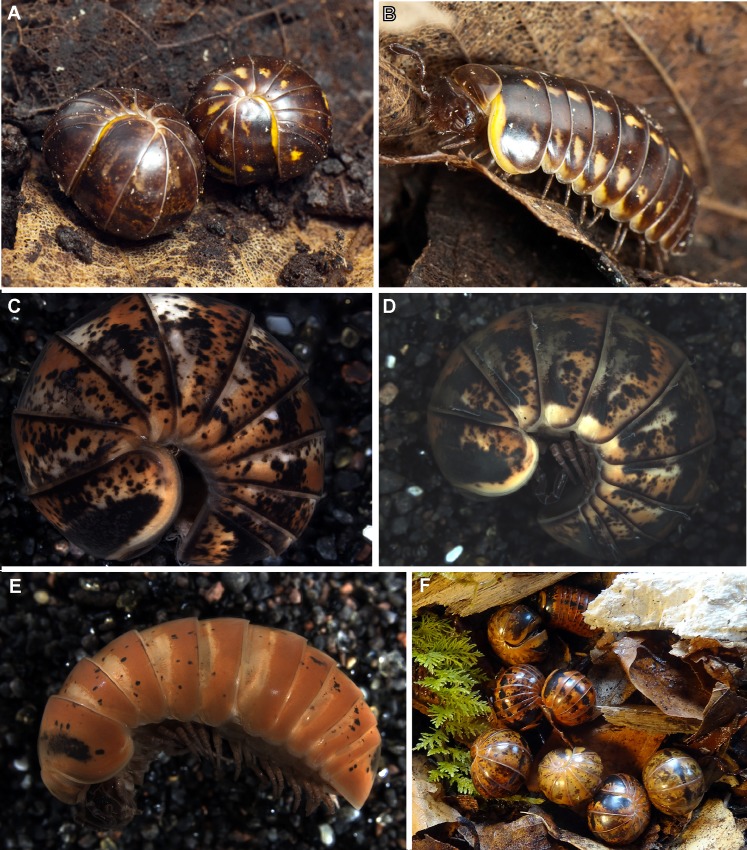
Different color morphs of *Glomeris klugii*. (**A)** Living, rolled-up specimens from Tübingen, SW Germany, left the 'undulata' morph, right the 'conspersa' morph, photograph courtesy of JP Oeyen; (**B)** Living, walking specimen from Tübingen, SW Germany, the 'conspersa' morph, photograph courtesy of JP Oeyen; (**C)** Specimen from Malgrate at the Lago di Como (ZFMK MYR4559); (**D)** Specimen from Malgrate at the Lago di Como (ZFMK MYR4767); (**E)** Specimen of *G*. *klugii porphyrea* from Croatia (ZFMK MYR734); (**F)** Population of *G*. *klugii porphyrea* from Croatia, photograph courtesy of P. Kautt.

**Fig 6:**
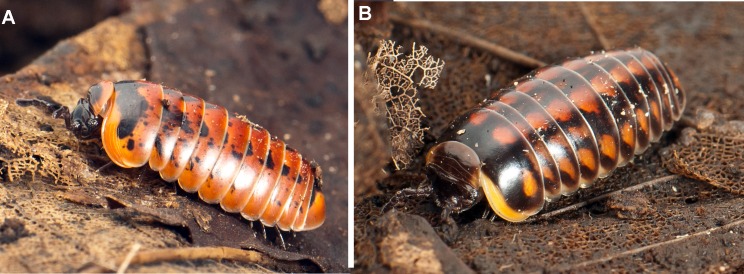
Different color morphs of *Glomeris klugii*. (**A)** Living, walking specimen from Austria, showing the red 'undulata' morph, photograph courtesy of JP Oeyen; (**B)** Living, walking specimen from Cremona, showing the rare red 'conspersa' morph, photograph courtesy of JP Oeyen.

No phylogenetic pattern could be discerned from the individual coloration pattern of the specimens (Fig 5A–5F). Even the same population often shows incredible individual variation in the color patterns (Fig 5A), but shows identical or almost identical haplotypes. The basic color of *G*. *klugii* is yellow with varying amounts of black freckles (e.g. [19]), while the Balkan subspecies *G*. *klugii porphyrea* is characterized by a red basic color. However, both yellow and red colored specimens of *G*. *klugii porphyrea* were collected from the same population in the Balkans (Fig 5E and 5F). The sequenced typically red colored specimens (Fig 5E) of *G*. *klugii porphyrea*, which closely resemble the holotype of *G*.*klugii* [20], clearly group within a clade of yellow colored Italian and German *G*. *klugii* (Fig 3). Characteristically red-black colored specimens also occur in Italy, such as in the population from Salo (Fig 6A), or even a red 'conspersa'-like from Cremona (Fig 6B), but do not group together (Fig 3). These observations confirm an earlier allozyme study [16] that found that specimens of the 'conspersa' color morph (Fig 5A right, 5B) are more closely related to specimens of the distinct 'undulata' color morph from the same locality (Fig 5A left) than to other 'conspersa' specimens from a different locality. Breeding experiments might reveal the hereditary basis of the development of different color patterns of *G*. *klugii*, or perhaps even show that the color patterns vary during different developmental stages of the same individual.

More Italian specimens of *G*. *klugii*, especially from currently unsampled areas such as the Piedmont or the Sea Alps, should be included in further studies to gain further insights in the intriguing haplotype diversity of *G*. *klugii* in Italy.

### Genetic distances of the COI barcoding fragment between the different *Glomeris* taxa

**Fig 7:**
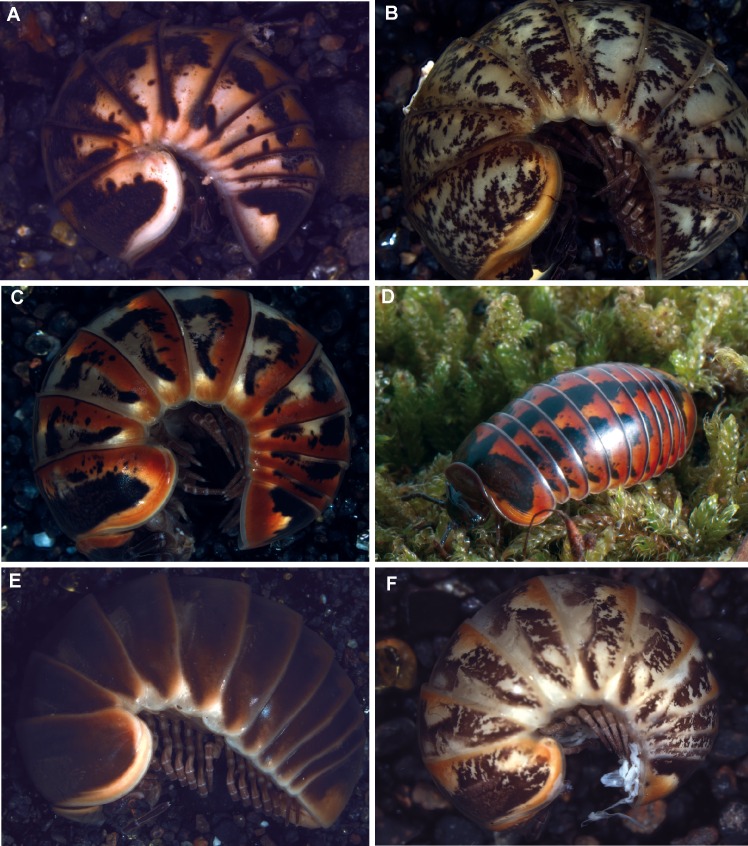
Different species of the *Glomeris klugii* species-group. (**A)** *Glomeris larii* Verhoeff, 1921 (ZFM MYR4562); (**B)** *Glomeris primordialis* Verhoeff, 1930 (ZFMK MYR4741); (**C)** *Glomeris oblongoguttata* Verhoeff, 1894 (ZFMK MYR4570) from Pisogne; (**D)** *G*. *oblongoguttata*, living specimen from Oltre il Colle, photograph courtesy of JP Oeyen; (**E)** *Glomeris oropensis* Verhoeff, 1936, adult specimen (ZFMK MYR4534); (**F)** *G*. *oropensis*, juvenile.

Aside from the distances observed between *G*. *larii* (Fig 7A) and *G*. *klugii* (0.2–4.4%), which fall clearly inside the intraspecific variation observed in *G*. *klugii* (0–5%, Fig 2), all other *Glomeris* species differ from one another by 6.9–15.9%. This degree of interspecific p-distances in the COI gene falls well within those observed in German ground beetles (3–20% [39]), but significantly lower than the interspecific distances observed in central European centipedes, which are between 16–19% in *Geophilus* [30] and 13.9–22% in *Cryptops* [41].

Closest to *G*. *klugii*, and falling inside the barcode gap of 5–8.5% otherwise observed between *Glomeris* species, is *G*. *primordialis*. *G*. *primordialis* is only known from two sites in the Piedmont area north of Biella [42,43] and has had no specimen records for 75 years. The sequenced specimens, while showing different haplotypes, all come from the same area (Table 1) and can be considered topotypes. Given the unique color pattern of *G*. *primordialis* (Fig 7B) and the genetic distance to *G*. *klugii*, a strong argument can be made to maintain the current classification as separate species. That said, more genetic studies involving Piedmont populations of *G*. *klugii*, which were not successfully included in the current study (Fig 1), should be conducted in the future.

The identity of *G*. *oblongoguttata*, a species endemic to the Bergamasque area with a color pattern comparable to the red morphs of *G*. *klugii* (Fig 7C and 7D), is confirmed as a separate species. The separation of *G*. *transalpina* and *G*. *klugii* was already supported by allozyme data [16] and is confirmed by our analysis.

**Fig 8:**
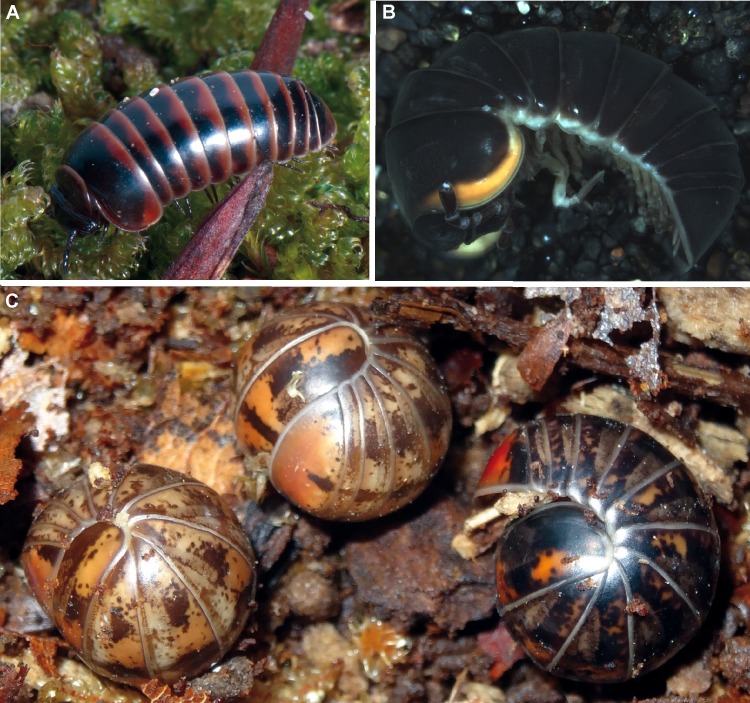
Different species of *Glomeris*. (**A)** *Glomeris transalpina* Koch, 1836, living specimen from the Riederalm, photograph courtesy of JP Oeyen (ZFMK MYR2609); (**B)** *Glomeris romana marinensis* Verhoeff, 1928 (ZFMK MYR797); (**C)** *Glomeris ligurica* Latzel, 1884, living specimens from a population close to Melogno, photograph courtesy of P. Kautt.

Morphologically, *G*. *oropensis* (Fig 7E), a high-altitude endemic currently only known from the mountain above Oropa [43], closely resembles *G*. *transalpina* (Fig 8A) as the species only differ in their juvenile coloration (Fig 7F). *G*. *oropensis* clearly differs genetically from the two nearby populations of *G*. *transalpina*, as well as from populations of *G*. *primordialis* and *G*. *klugii*. The observed large genetic distances confirm that *G*. *oropensis* is currently best viewed as a separate species, microendemic to the Biellese Alps.

Our analysis also confirms earlier allozyme studies [17] that *G*. *transalpina* (Fig 8A) is a species clearly separate from, but close to, *G*. *klugii*. Interestingly, the sequenced specimens of *G*. *transalpina* originating from two separate high-altitude populations (Riederfurka and Simplon, Fig 1) show identical haplotypes, hinting to a recent dispersal (from an ice age refugium?) of the species to both localities.

*G*. *romana* and *G*. *klugii* are clearly separate genetically despite their morphological similarity (both species show a unique color pattern produced by varying densities of black freckles) and geographical exclusiveness (*G*. *romana* occurs further south than *G*. *klugii*, both species apparently never occur in direct sympatry [6,14]). This confirms again results of earlier allozyme studies [6,8]. The present study provides no molecular phylogenetic support for the melanistic subspecies *G*. *romana marinensis* Verhoeff (Fig 8B) described from San Marino (which shares the absence of any main stria on the thoracic shield with *G*. *klugii*).

All other analyzed species, *G*. *marginata*, *G*. *connexa*, *G*. *apuana* and *G*. *ligurica* differ clearly from one another and from the species of the *G*. *klugii* species group. Intraspecific distances of all species aside from *G*. *klugii* are much lower than in *G*. *klugii*, 0–2.5% *vs*. 0–5%. One possible explanation for this difference might lie in the fact that this study only included a few samples for the other species and that these samples do not cover the geographical extremes of their specific range [6]. This is a contrast to *G*. *klugii*, a focus of this study, for which the specimens were collected from 18 different localities representing the entire geographic range of the species (Fig 1).

### *G*. *larii*, a new synonym of *G*. *klugii*

*G*. *larii* was described by Verhoeff as a species similar to, but separate from *G*. *klugii* [44]. The main argument for the species status of *G*. *larii*, despite its great similarity to *G*. *klugii* in all morphological aspects, was the color pattern of the anal shield (Fig 7A). Furthermore, *G*. *klugii* and *G*. *larii* were supposed to be geographically exclusive, with only *G*. *larii* occurring on the eastern coast of the Lago di Como around Lecco. *G*. *larii* was only collected twice; the type series and a second batch of specimens a few years later [45]. Aside from these two occasions, there are no further records of *G*. *larii* and the species is only mentioned in faunal lists [6,9,10].

Here, we document the color variability of *G*. *klugii* (Fig 5A–5F), which encompasses the color pattern observed in *G*. *larii* (Fig 7A). *G*. *klugii* also regularly occurs around Lecco (own observations, collections of the Bergamo Museum). Our own sample from a locality very close to Lecco includes specimens with the color pattern of *G*. *larii* (Fig 7A) and specimens with the color pattern more associated with *G*. *klugii* (Fig 5C and 5D). These specimens all group together, well within *G*. *klugii*, in the COI and combined analyses (Figs 3 and 4).

The genetic distances between *G*. *larii* and *G*. *klugii* fall well within the intraspecific variation observed in *G*. *klugii* and other *Glomeris* species (Fig 2). Based on the combination of (1) no geographical separation (direct sympatry, (Fig 1)), (2) no reliable differences in their morphology and coloration (Figs 5C, 5D and 7A), and (3) genetic distances of both mitochondrial and nuclear genes well within the intraspecific distances observed in other *Glomeris* species (Fig 2), the name *G*. *larii* is best viewed as being synonymous with *G*. *klugii*.

### The status of *G*. *genuensis*

Our attempts to separate genetically specimens of *G*. *genuensis* from *G*. *ligurica* failed; all sequenced specimens grouped with previously sequenced *G*. *ligurica*. Even specimens collected at the type locality of *G*. *genuensis*, both showing the 'freckled' look associated with *G*. *genuensis* and the *connexa*-look associated with *G*. *ligurica* (similar to Fig 8C) show identical haplotypes. Authors who recollected *G*. *genuensis* always describe key morphological characters, the striae and the anal shield coloration, as identical to those known from *G*. *ligurica*. One population of *G*. *genuensis* was even described as a variety aptly named 'pseudoligurica' [42] because of the great similarity to specimens of *G*. *ligurica*.

All recorded specimens of *G*. *genuensis* and *G*. *ligurica* [12,13,44,46,47] show identical striated patterns on their thoracic shield, an important taxonomic character. A direct comparison of their colors indicates that both species also share similar unique characteristics on their anal shield. In fact, the main difference between *G*. *genuensis* and *G*. *ligurica* seems to be in the more freckled color pattern of the former, This difference is similar to the characters used to justify the distinctiveness of *G*. *conspersa* and *G*. *undulata*, now both known as color variants of *G*. *klugii* [14,16]. Currently, *G*. *genuensis* and *G*. *ligurica* appear to be morphologically and genetically inseparable. However, further studies involving more sample localities and specimens of Ligurian *Glomeris* are necessary to evaluate the status of *G*. *genuensis* and formally synonymise the name under *G*. *ligurica*.

### Phylogeny of the *Glomeris* species

Our choice of molecular markers is not the best to resolve the phylogeny of the different species of *Glomeris*. The chosen 28S rDNA fragment [31] was too conservative for our dataset, providing little to no resolution. A different 28S rDNA fragment might work better for genus-level studies of the Glomerida, but such a gene has not been explored so far in the order, or even in Diplopoda in general. The mitochondrial COI gene provided surprisingly good resolution in our phylogeny on the species-level (Fig 3), as well as for the relatively closely related *G*. *klugii* and the *G*. *connexa* species-groups. However, the relationships between the different *Glomeris* species is still little resolved. As a mitochondrial marker, which is only inherited from the maternal line, interpretation of the COI results has to be taken with care. Mitochondrial markers are prone to show high variability because of the presence of inherited endosymbionts [48]. However, *Wolbachia* infections have not yet been recorded in the Diplopoda. Additional risks of interpreting the results of studies depending on mitochondrial molecular markers are gene transfers (e.g. [49–51]) as well as sex-dependent dispersal strategies, in which males are much more mobile then females [52]. While both events have not yet been recorded in the Diplopoda, the still small number of genetic studies in millipedes [2], in combination with our generally limited knowledge of the ecology and life history of pill millipedes [14], demands a careful interpretation of the obtained results.

## Conclusion

*G*. *klugii* exhibits large intraspecific distances of up to 5% across its area of distribution, especially among Italian populations. Individuals of *G*. *klugii* belonging to the same color morphs do not form phylogenetically related groups. A clear geographic distribution pattern of the different Italian haplotypes cannot be discerned in our dataset. Only distinctly related populations of *G*. *klugii* occur in close proximity to one another.

*G*. *larii* is synonymous to *G*. *klugii*. The other studied local Italian endemics, *G*. *primordialis* and *G*. *oropensis* both microendemics in the Biellese Alps, as well as *G*. *oblongoguttata* endemic to the Bergamasque Alps, are, despite their morphological similarity to *G*. *klugii*, supported as separate species. The barcoding COI fragment is a useful tool to identify difficult to determine pill millipede species.

The results of our study show how important integrative taxonomic studies are to distinguish between 'endemic' species that exist only as names in the literature ([16,27] *G*. *larii* this study) and 'real' local endemic species ([11,17] this study) before any biogeographical interpretations are undertaken. Our analyses confirm that northern Italy, especially the Biellese and Bergamasque Alps, is indeed unusually rich in local endemic pill millipede species. The evolutionary processes that led to such local endemism remain unknown. A correct aging of the splits between the different *Glomeris* taxa might elucidate whether these local endemic species evolved relatively recently, i.e. during the last ice ages, or whether far older, geological processes might have shaped the current center of endemism patterns in northern Italy. Future research on these evolutionary processes that will include local endemics that are not pill millipedes is planned.

## Supporting Information

S1 Alignment Fasta alignment combined COI & 28S dataset. (FAS)

S1 Table Pairwise distances matrix. (XLS)
